# Language of the Hand

**Published:** 1887-07

**Authors:** Joseph Simms


### LANGUAGE OF THE HAND.

By Joseph Simms, M. D.

Hands indicate character. A thin, skinny, narrow palm expresses feebleness of intellect, as well as absence of energy or moral force. A hollow, deep palm indicates misfortune, loss of money, misery and failure in enterprises. Shakespeare tells of an "itching palm:" that indicates that the blood is out of order, with a covetous disposition. A stiff, hard hand, that opens with difficulty to its full extent, betrays stubbornness of character and reluctance to open to calls of charity. Supple elastic fingers, on the other hand, while manifesting a tendency to extravagance, nevertheless indicate talent and sagacity. Those who have short fingers are quick, impulsive, and act usually on the spur of the moment, more readily than those who have long fingers. Short, thick fingers, nearly all of the same length, indicate a callous, cruel character, and betray clumsy unhandiness in manipulation, as well as a constant tendency to falsehood and the defamation of the character of others. Long, slender fingers betray a peevish, worrying disposition. Young women ought to choose a husband whose hands are naturally red; and hands made red with difficulty should be carefully avoided. A man with dark colored hands is inclined to biliousness and melancholy. As an indicator of character, however, the thumb is the "boss." A small, ill-formed, feebly-balanced thumb betrays a vacillating disposition. Small thumbed persons are governed by the heart, while the large thumbed are swayed by the intellect. Independent, self-reliant people have large thumbs, or ought to have them, from the point of view of the chirosophist, while pliant, dependent, and easily governed natures may be known by the smallness of that digit, always remembering that the feature must be judged in proportion to the size of the hand and the fingers on the same hand.

